# Editorial: New insights in prosthetic joint infections

**DOI:** 10.3389/fmicb.2024.1522400

**Published:** 2024-11-27

**Authors:** María Guembe, Nils P. Hailer, Pablo Sanz-Ruíz

**Affiliations:** ^1^Department of Clinical Microbiology and Infectious Diseases, Hospital General Universitario Gregorio Marañón, Madrid, Spain; ^2^Instituto de Investigación Sanitaria Gregorio Marañón, Madrid, Spain; ^3^Department of Surgical Sciences/Orthopaedics, Uppsala University Hospital, Uppsala, Sweden; ^4^Department of Orthopaedic Surgery and Traumatology, Hospital General Universitario Gregorio Marañón, Madrid, Spain; ^5^Medicine Department, School of Medicine, Universidad Complutense de Madrid, Madrid, Spain

**Keywords:** prosthetic joint infection, PJI, Biofilm, arthroplasty, debridement, diagnostic strategies, antibiotics

Prosthetic Joint Infection (PJI) is an important healthcare problem which affects 1%−2% of patients undergoing prosthetic surgery, having become the most common reason for early revisions after knee arthroplasty and the second most common reason after hip arthroplasty. PJI causes enormous suffering, it is associated with a mortality being higher than in some cancer, such as prostate or breast malignancies, and costs for healthcare often exceed 100,000 € per affected patient. The main causative and differentiating factor related to PJI is the microorganisms' ability to form a biofilm, which confers high resistance to antibiotics and to host immune response. Thus, this implies a difficulty in combating PJI, the ineffectiveness of many antibiotics and, in most cases, the need for prosthesis exchange. Despite recent advances in prevention and treatment, there is still an urgent need to enhance our basic understanding of the pathophysiology and to improve clinical success rates. Different surgical treatments have been proposed, such as radical debridement, one or two stage or even resection arthroplasty, combined with different antibiotic protocols (short duration, long duration, suppression treatment…). Recent studies show success rates of around 70% with current treatment strategies. In the current Research Topic, six original articles, including perspectives and reviews, have been published toward new approaches in diagnosis and treatment of PJI.

Bogut et al. undertake the genomic characterization of a small colony variant (SCV) strain of *Staphylococcus epidermidis* derived from a patient with a late PJI. The authors hypothesize that multiple deletions, amongst them the absence of the Staphylococcal cassette chromosome, may contribute to switching of this bacteria's phenotype to a SCV, and to streamlining the genetic content toward adaptation to chronicity. Interestingly, in the reported strain, some single nucleotide polymorphisms found in genes related to virulence factors and biofilm formation may also be associated with the development of SCV. Surely, in the near future, genomic analysis of bacteria isolated from PJI patients will help physicians understand the virulence, resistance patterns and the propensity to form biofilm. This will enable improved, personalized antibiotic regimes and facilitate the choice between more conservative, implant-preserving and more aggressive surgical strategies.

The diagnosis of PJI remains challenging, and numerous approaches to increase the accuracy of analyses of joint fluid aspirates have been undertaken. In the paper by Maritati et al., the activity of the leukocyte-derived enzyme myeloperoxidase is measured in such aspirates from patients with or without PJI, and the diagnostic accuracy is evaluated and compared to the established leukocyte-esterase assay. By calculating an area under the curve (AUC) of 0.86 and a cut-off value discriminating between infected and non-infected patients, the authors find good diagnostic accuracy and propose that the assay be validated in larger samples.

In the effort to improve diagnosis through synovial fluid aspiration, Morovic et al. analyze the presence of D-lactate and L-lactate as potential biomarkers for PJI. This approach is based on the fact that bacteria and fungi are uniquely capable of producing both forms of lactate, whereas human cells can only produce the L-lactate isoform. To test this hypothesis, they analyzed D-lactate and L-lactate concentrations in cultures of the main bacteria responsible for PJI. They observed that all bacterial strains produced D-lactate (regardless of whether they were in planktonic or biofilm form), unlike L-lactate, which was only produced by certain strains. Consequently, they recommend D-lactate as a more reliable biomarker than L-lactate.

Unfortunately, it is sometimes impossible to obtain synovial fluid for analysis, highlighting the need for serum biomarkers to improve PJI diagnosis. In the study by Lu et al., the authors evaluate the efficacy of serum protein electrophoresis (SPE) for PJI diagnosis. They analyze different serum proteins (C-reactive protein, erythrocyte sedimentation rate, D-dimer, fibrinogen, serum albumin, and serum protein proportion) using SPE in two groups of patients undergoing prosthetic revision surgery, one for aseptic reasons and the other for septic causes. Among the biomarkers analyzed, the authors propose alpha-1 globulin as a potential biomarker, showing an AUC of 0.86, comparable to that obtained for CRP and ESR, thus recommending its combined use.

Regarding approaches to improve patient outcome, it is needed to deeply assess new strategies and alternatives capable of eradicating sessile cells embedded in the biofilm when a PJI has been established. Ferreira et al. reviewed the evidence of biofilm-active drugs and the new alternatives to classical regimens for PJI highlighting the importance of rifampicin resistance and that it should not be prescribed as monotherapy. Moreover, it is essential to choose initial regimen of combined antibiotics based on anti-biofilm ability, with a minimal risk of resistance development, and with adequate bone absorption. Regarding total duration of antibiotic therapy and for how long the initial IV regimen should be administered, is still under discussion.

It is well known that irrigation with antiseptic solutions is a critical step in the treatment of PJI, but there is no consensus of which antiseptic and regimen to use. In the perspective of Valdés and Minter, not only potential insights of a novel citrate-based irrigation solution showed promising results for the treatment of PJI, but also as for its prevention. Despite findings were based on *in vitro* experiments, citrate-based solutions showed increased antimicrobial properties, greater biofilm disruption, increased exposure time, and reduced cytotoxicity compared to conventional solutions. However, future studies are needed to calculate minimum biofilm eradication concentration and to validate its utility in more large-scale *in vivo* analysis.

The current Research Topic thus highlights important aspects around both diagnosis and treatment of PJI, contributing valuable insights to this challenging clinical problem.

